# Supplementary material for: Predictors of Death in Patients with Neonatal Sepsis in a Peruvian Hospital
Source: Trop Med Infect Dis. 2022 Oct 31;7(11):342. doi: 10.3390/tropicalmed7110342 (PMC9697646; doi:10.3390/tropicalmed7110342)
Supplement: Supplementary file 1 [file tropicalmed-07-00342-s001.zip › tropicalmed-1886131-supplementary.pdf]

Supplemental Table S1: Cox 1 Regression for Predictors of Mortality in Early-Onset Sepsis

Variable	cHR (95% CI)	p-Value	aHR (95% CI)	p-Value
Gestational age				
- Term newborn (>37)	Ref		Ref	
- Preterm newborn (<37)	33.47 (11.80-94.89)	<0.001	33.11 (2.66-411.85)	0.007
Leukocytes (cells/mm3)				
- Normal (<12000)	Ref		Ref	
- Leukocytosis (>12000)	0.41 (0.22-0.79)	0.007	1.69 (0.48-5.86)	0.412
Platelets (cells x 103/L)				
- Normal (>150000)	Ref		Ref	
- Low count (<150000)	3.48 (1.86-6.52)	<0.001	8.04 (1.68-38.55)	0.009
Total Immature Index (IIT)				
- Normal (<0.12)	Ref		Ref	
- High count (>0.12)	11.63 (2.75-49.07)	0.001	-	-
Glucose (mg/dL)				
- Normal (<150)	Ref		Ref	
- High count (>150)	4.75 (2.26-9.97)	<0.001	0.39 (0.09-1.79)	0.227
Creatinine (mg/dL)				
- Normal (<1.1)	Ref		Ref	
- High count (>1.1)	3.08 (1.42-6.66)	0.004	0.77 (0.22-2.79)	0.696
pH				
- Normal (>7.35)	Ref		Ref	
- Low count (<7.35)	2.76 (0.94-8.14)	0.066	2.44 (0.33-18.19)	0.384
CO2 pressure (mmHg)				
- Normal (<45)	Ref		Ref	
- High count (>45)	2.14 (0.94-4.91)	0.071	0.76 (0.21-2.70)	0.668
PaO2/FiO2*				
- Normal >400	Ref		Ref	
- ARDS<400	6.45 (0.86-48.26)	0.070	1.18 (0.10-13.51)	0.892

cHR: crude Hazard ratio, aHR: adjusted Hazard ratio, PaO2/FiO2: fraction between arterial oxygen pressure over the inspired fraction of oxygen, Ref: reference group.

Supplemental Table S2: Cox 2 Regression for Predictors of Mortality in Early-Onset Sepsis

Variable	cHR (95% CI)	p-Value	aHR (95% CI)	p-Value
Birth weight				
- Normal (>2500 grams)	Ref		Ref	
- Low birth weight (<2500 grams)	19.67 (8.18-47.32)	<0.001	3.63 (1.11-11.83)	0.032
APGAR score at 1 minute				
- Normal	Ref		Ref	
- Moderate	4.37 (2.09-9.13)	<0.001	0.57 (0.25-1.35)	0.208
- Low	3.34 (1.44-7.69)	<0.001	0.47 (0.16-1.41)	0.180
APGAR score at 5 minutes				
- Normal	Ref		Ref	
- Moderate	3.40 (1.76-6.58)	<0.001	2.77 (1.15-6.64)	0.023
- Low	-	-	-	-
Septic Shock				
- No	Ref		Ref	
- Yes	14.22 (7.51-26.91)	<0.001	2.72 (1.25-5.93)	0.012
ARDS				
- No	Ref		Ref	
- Yes	19.99 (7.75-51.53)	<0.001	2.83 (0.79-10.12)	0.109
Use of IMV				
- No	Ref		Ref	
- Yes	21.52 (8.34-55.55)	<0.001	4.39 (1.35-14.23)	0.014
Use of CVC				
- No	Ref		Ref	
- Yes	1.56 (0.21-11.56)	0.665	2.06 (.020-21.00)	0.541
Use of exclusive breastfeeding				
- No	Ref		Ref	
- Yes	0.47 (0.25-0.90)	0.023	0.25 (0.12-0.54)	<0.001

cHR: crude Hazard ratio, aHR: adjusted Hazard ratio, ARDS: acute respiratory distress syndrome, IMV: invasive mechanical ventilator, CVC: central venous catheter, Ref: reference group.
